# Laparoscopic treatment of large bowel obstruction due to a self-locating peritoneal dialysis catheter

**DOI:** 10.1016/j.ijscr.2018.10.069

**Published:** 2018-11-02

**Authors:** Tamara Díaz Vico, José Luis Rodicio Miravalles, Emilio Sánchez Álvarez, María Moreno Gijón, Amaya Rizzo Ramos, Estrella Olga Turienzo Santos, Lourdes Sanz Álvarez

**Affiliations:** General Surgery, Hospital Universitario Central de Asturias (HUCA), Spain

**Keywords:** Peritoneal dialysis, Catheter, Intestinal obstruction, Laparoscopic approach, Case report

## Abstract

**Introduction:**

Peritoneal dialysis has been used in the treatment of end-stage renal disease for a long time. The development of continuous ambulatory peritoneal dialysis (CAPD) has achieved an acceptable device of renal replacement therapy.

**Presentation of case:**

We report a 55 year-old patient who was initiated on CAPD in February 2016. Three months later, the Tenckhoff catheter was removed due to its malfunction, and a new self-locating peritoneal dialysis catheter was placed in the left side of the abdomen. In September 2016, the patient presented with symptoms of intestinal obstruction. A CT scan revealed a collapsed sigmoid colon with the tungsten tip of the catheter supported on the mesosigmoid as the cause of the occlusion.

**Discussion:**

Herein, a rare but clinically important case of mechanical large bowel obstruction due to self-locating peritoneal dialysis catheter is presented. The weight added to the tip of the self-locating catheter for the purpose of stretching it, can be dangerous if a displacement takes place. A laparoscopic procedure was performed, resolving the obstruction by reinserting the peritoneal catheter in its right position.

**Conclusion:**

The weight added to the tip of self-locating catheters is a matter of concern, since intimate contact between the peritoneal catheter and the intestinal wall can result in perforation or intestinal occlusion.

## Highlights

• Intestinal obstruction due to self-locating peritoneal dialysis catheters is an infrequent condition.
• A 55-year-old patient diagnosed with large bowel obstruction was successfully treated by laparoscopic approach.
• The weight added to the tip of self-locating catheters can cause different complications, including decubitus ulcers or perforations of soft tissues.

## 1 Introduction

This work is reported in line with the Surgical Case Report Guidelines (SCARE) [1].

Chronic kidney disease (CKD) in the population aged 65–74 years in European countries varies from 4.1% to 25.5% [2]. In 1976, Popovich et al introduced the concept of continuous ambulatory peritoneal dialysis (CAPD). Now, peritoneal dialysis (PD) has shown to be an effective method of renal replacement therapy.

Since Tenckhoff described the permanent silicone peritoneal catheter fifty years ago [3], a wide variety of new models with functional modifications have been designed [4].

The first models were based on the classical Tenckhoff design, with variations in the number or shape (polyester fiber, disc-ball, etc.) of the cuffs, in the design of the tip (straight, coiled), and in the shape of the subcutaneous portion (straight or with a permanent bend); ”named” models, with variations mainly in the intraperitoneal segment (the T-fluted Ash, the Lifecath, the Toronto-Western, the Ronco, and the Valli catheters) or materials (the Cruz catheter); and new models, also based on the Tenckhoff design, but with functional modifications, such as the Vicenza “short” catheter (with a shorter intraperitoneal segment for a lower insertion that prevents dislocation and improves body image acceptance) or the Di Paolo self-locating catheter, with tungsten attached to the tip for keeping it firmly in the Douglas pouch [3,5].

The evolution and innovation of peritoneal dialysis catheters pretend to achieve a decrease in the overall complication rate. However, complications after dialysis catheter implantation are still a matter of concern and, acute intestinal obstruction is, by no means, a common complication [6].

## 2 Presentation of case

We report a 55-year-old male patient, without additional surgical background, who had end-stage renal disease due to IgA glomerulonephritis. While waiting for kidney transplant surgery, the patient initiated continuous ambulatory peritoneal dialysis (CAPD) in February 2016, using a Tenckhoff catheter, which was flushed regularly once a week and was functional during the first three months after insertion. This catheter, placed in the right side of the abdomen, was removed three months later due to its malfunction, and a new self-locating catheter was placed in the left side. However, the new device failed to achieve the optimal functionality.

In September 2016, the patient presented with features suggestive of intestinal obstruction: vomits, abdominal distension, and no gas passing for 48 h. On physical examination, he became dehydrated, and abdominal examination showed distension, with tenderness in the upper abdomen, and metallic bowel sounds.

**Fig. 1:**
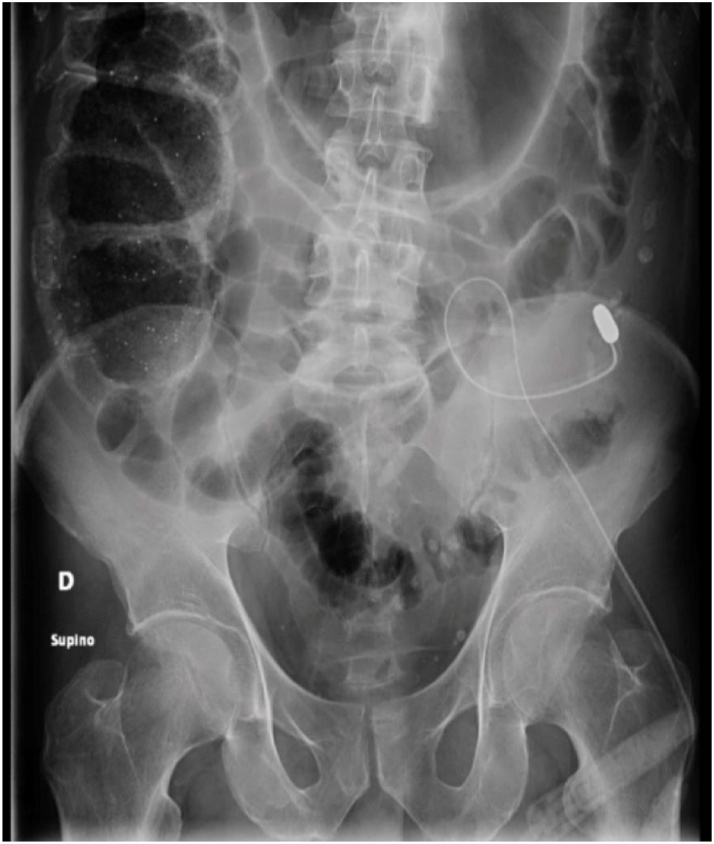
X-ray showing the tungsten tip of the catheter in the left iliac fossa, with intestinal distension.

A plain X-ray of the abdomen was performed to identify the catheter tip position, which appeared to be in the left iliac fossa, instead of the central part of the pelvic cavity. Also, important distension of the right colon and bowel loops were observed (Fig. 1). A decrease in effluent volume due to catheter displacement was a consequence of the bowel entrapment. Contrast-enhanced CT abdomen and pelvis showed a collapsed sigmoid colon with the tungsten tip of the catheter supported on the mesosigmoid. Contrast enema was also performed, with no evidence of contrast extravasation. No ascites or inflammatory fluid collection was observed (see Supplemental Digital Content, multimedia, available at https://www.youtube.com/watch?v=NRUKICmx8qY).

The patient underwent an urgent exploration of the peritoneal cavity. Laparoscopic approach was made using two 12 mm and one 5 mm trocars. The tungsten tip of the catheter was adhered to the mesosigmoid and, as a consequence, a progressive sigmoid obstruction occurred. No evidence of perforation or peritonitis was observed. A special emphasis was placed on the wall of the colon and, once we confirmed the absence of serosal tears, the catheter tip was mobilized and placed in the Douglas pouch, achieving a decompression of the obstructed bowel.

In order to stretch the self-locating catheter and avoid future recurrences, we used non-absorbable suture to fix it at the parietal peritoneum of the pelvis.

**Fig. 2:**
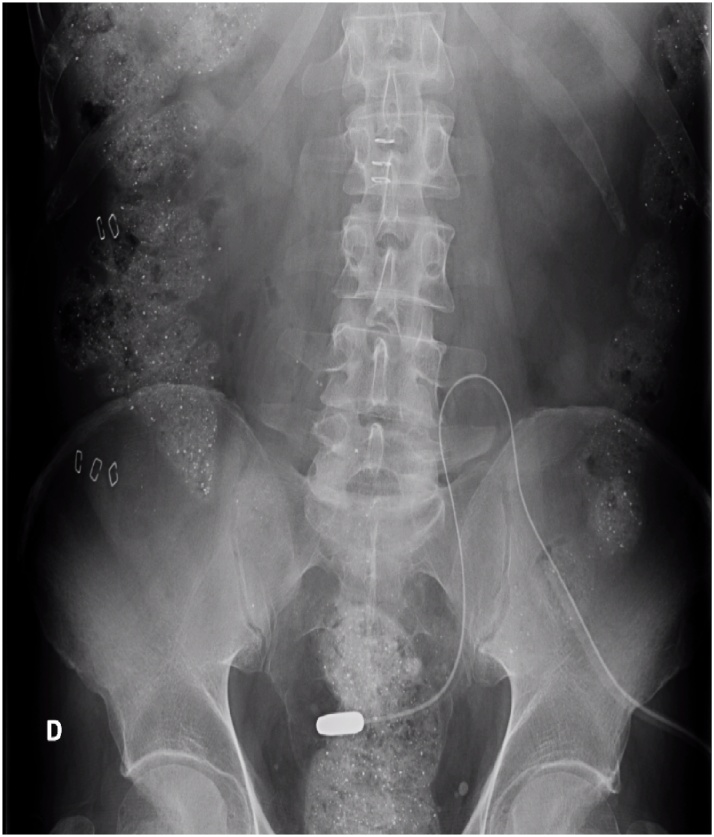
Tungsten tip of the catheter in its right position, with no evidence of intestinal obstruction.

After resolving the obstruction by reinserting the peritoneal catheter in its right position (Fig. 2), the patient recovered well postoperatively, tolerating oral intake 24 h later. He was discharged on the third postoperative day. The catheter remained functional after eight-month follow-up, when the patient underwent surgery for kidney transplant.

## 3 Discussion

Modifications of the CAPD technique, with its developing advances and easier modalities, have made this technique a widely accepted form of renal replacement therapy.

Complications after dialysis catheter implantation can be categorized as early or late. Early complications are those that occur within 30 days after implantation, as peritonitis, catheter malfunction, leak, and perforated viscus. Late complications include infections, catheter migration, and hernias [6].

Acute bowel obstruction is a common abdominal emergency. The majority of cases occur secondary to adhesions from prior surgery. We found no previous references in the literature to large bowel obstruction caused by peritoneal dialysis catheter, nor to the laparoscopic approach for catheter reposition.

The weight added to the tip of the self-locating catheters to straighten it can be dangerous if a displacement takes place [7]. The suggested mechanism is that the peritoneal dialysis catheter tip migrated to the left iliac fossa and the added weight favored the occlusion of the sigmoid colon. When the catheter tip was mobilized, the colon was then released and returned back progressively to its normal position.

Other designs and innovations have been proposed to reduce catheter-related complications, including variations in the number or shape of the cuffs, design of the tip and subcutaneous portion, materials of the intraperitoneal segment, or new models as the self-locating catheter, with tungsten attached to the tip for keeping it firmly placed in the Douglas pouch.

To the best of our knowledge, minimally invasive techniques can be considered for the management of bowel obstruction. In selected patients and with appropriate skills, laparoscopic approach can be attempted and may be beneficial, resulting in a faster recovery [8,9].

## 4 Conclusion

The weight added to the tip of self-locating catheters is a matter of concern, as they can cause decubitus ulcers or even perforations of soft tissues [10] as, for instance, the intestinal wall. Despite the development of CAPD, and the improvements achieved in peritoneal dialysis devices, we believe these potential risks have to be taken into account when using self-locating peritoneal dialysis catheters.

## Conflicts of interest

There are no conflicts of interest.

## Funding source

No funding source.

## Ethical approval

Case reports do not require ethical approval by our institution if both patient identification and clinical case are anonymous.

## Consent

- Written informed consent was obtained from the patient for the publication of this case and the accompanying images.
- We state that the work has been reported in line with the PROCESS criteria.

## Author contribution

- **Tamara Díaz Vico**: Reviewed the literature and wrote the article.
- **José Luis Rodicio Miravalles**: reviewed the paper and final editing of the article.
- **Emilio Sánchez Álvarez**: reviewed the paper.

## Registration of research studies

NA.

## Guarantor

Tamara Díaz Vico.

José Luis Rodicio Miravalles.

## Provenance and peer review

Not commissioned, externally peer reviewed.
